# A Fast Bubble Detection Method in Microtubes Based on Pulsed Ultrasound

**DOI:** 10.3390/mi12111402

**Published:** 2021-11-15

**Authors:** Yiqing Li, Junwu Wu, Leijie Fu, Jinju Wang

**Affiliations:** 1 School of Mechatronic Engineering, Xi’an Technological University, Xi’an 710021, China; j.wu@softdynamics.org (J.W.); fuleijie@xatu.edu.cn (L.F.); 2 School of Mechanical Engineering, Xi’an Jiaotong University, Xi’an 710049, China; j.wang@softdynamics.org

**Keywords:** pulsed-ultrasound, microbubble, two-phase flow, flow gauging accuracy

## Abstract

In the process of biological microfluidic manipulation, the bubbles generated in the tube will seriously reduce the gauging accuracy. This paper introduces an improving method that can estimate the size of microbubbles in real time. Hence, the measurement data of the liquid volume can be modified according to this method. A microbubble detector based on the pulsed-ultrasound method was studied, including the device structure and the working principle. The assessment formula of the microbubbles in the tube was derived from the simulation results, which adopted the two-phase theory. The digital image processing method was applied to fulfill the microbubble calibration. This detection method was applied to measure the microbubbles in the tube and to modify the flow volume in a timely manner. The results of the experiments showed that this method is effective at improving the microflow gauging accuracy.

## 1. Introduction

In clinical medicine and biological research, we often encounter situations requiring accurate fluid measurement, such as quantitative blood transfusion, biological microfluidic sample testing, etc. [1]. The bubbles in the flow undoubtedly disturb the fluid action, especially in the case of microfluidic processing. The bubbles can obstruct the semipermeable membrane in blood dialysis, disorder the indexes in the blood-gas analysis, and even put a patient under transfusion in danger [2,3]. In biomedical microfluidic experiments, the bubbles existing in the tube will seriously reduce the measurement accuracy. However, whether bubbles are generated depends on many factors, such as the pumping actuation, pressure differences along their path, different pressures between the inside and outside of the liquid surface, etc., so they are difficult to avoid and eliminate [4,5].

Although it is difficult to eliminate bubbles in the flow, it is possible to measure and control them [6]. Especially in microflow gauging, the bubble size and velocity are monitored in real time and compensated for in the fluid volume, which is a relatively effective method. Many technologies are currently adopted for the detection of microbubbles in tubes, such as capacitive detection [7], photoelectric detection [8], ultrasonic detection [9], image processing [10,11], etc. For capacitive detection, a high detection accuracy is difficult to obtain, and the photoelectric detection method is affected by the liquid’s color.

In recent years, due to the development of machine-learning techniques, bubble-detection methods using image processing have been widely studied, and such methods are able to obtain good results [12,13]. However, ultrasound is still a good choice to detect the bubbles in microtubes because of its good directional capability, penetrating ability, easy acquirement, greater accuracy, etc. Ultrasound techniques for bubble detection in flows can be classified into three groups: transmission techniques, Doppler techniques, and pulse techniques [14]. The transmission technique can measure bubble sizes. However, this technique has a low accuracy and position resolution. The Doppler technique is suitable for velocity field measurement, but is less used to measure the bubble size. The pulse technique measures the axial (along an ultrasonic beam) distance between the ultrasonic transmitter and a receiver or a reflecting object [15]. Applying the pulsed-ultrasound technique, the sizes and velocities of microbubbles in the flow in the tube can be obtained in real time. The detection process is not affected by the liquid color, nor the material of the microtubes.

In this paper, a fast bubble-detection method based on ultrasound technology is proposed. To verify the effectiveness of the method, a bubble detector was fabricated. This detector was applied to measure microbubbles in the tube and to modify the flow volume in a timely manner. The results of the experiment showed that the proposed method is effective at improving the microflow gauging accuracy.

## 2. Materials and Methods

### 2.1. The Principles of Bubble Movement in Microtubes and the Ultrasound Probe

The movement of a bubble with the flow in a microtube is different from that in a macrotube. If we observe microbubbles in the flow, there is a clear interface between the bubbles and the liquid. We considered the liquid as a continuous phase and the bubbles as a disperse phase. Adopting the FLUNT software, we built a traceable volume of fluid (VOF) model of the two-phase (gas–liquid) interface and studied its movement properties. From the simulation, we knew that during the process, the bubble shape had a slight change, similar to an ellipsoid, because of the effect of surface tension and viscous drag. The flow velocities of the bubble and liquid were almost equal if we kept the injection rate constant. The conclusion of the simulation corresponded to the observation experiment. The flow movement characteristics showed that the ultrasound was adapted to detect the microbubbles in the flow. The designed detection system was based on the ultrasonic transmission-medium-attenuation principle. Ultrasonic attenuation can be classified into three types: diffusion attenuation, absorptive attenuation, and scattering attenuation. The bubbles in the microtube would lead to the latter two attenuations. If the ultrasonic plane wave normal traverses the interface of the liquid–gas phase, its energy would be reflected and transmitted. We assumed the characteristic sound impedance of two phases as $Z_{1}$ and $Z_{2}$. The sound intensity reflection ratio *R* and sound intensity transmission ratio *T* are defined as:

(1) $$R={\left(\frac{Z_{2}-Z_{1}}{Z_{2}+Z_{1}}\right)}^{2}$$

(2) $$T=\frac{4Z_{1}Z_{2}}{{\left(Z_{1}+Z_{2}\right)}^{2}}$$

The characteristic acoustic impedance of water and air are $1.5\times 10^{6}$ kg/m${}^{2}$s and 430 kg/m${}^{2}$s, respectively. According to Equations (1) and (2), if the ultrasonic wave is incident from water to air, their interface sound intensity reflection ratio *R* is 0.999, and the sound intensity transmission ratio *T* is 0.001. This means that when the ultrasonic wave is incident from water to air, almost all of its energy is reflected on the boundary surface. In the detection system, we launched a high-frequency ultrasound pulse wave on one side of a pipeline with flow, and the wave through the pipeline was received on the other side. Once the bubbles appeared in the flow, the received wave energy decreased significantly because of absorptive attenuation and scattering attenuation. If the bubbles are large enough, ultrasound can only diffract at the bubbles’ edges and form an acoustic shadow. Most ultrasonic energy is absorbed by the bubble, so the received signal amplitude would be reduced to a very low value, and the received signal frequency would change. In the signal-processing circuit, adopting D/A conversion and signal interruption, we can obtain the variation in time of the ultrasonic signal frequency and voltage amplitude. Thus, we can obtain the interval time Δt, which is the bubble going through the ultrasonic probe [16]. Because the speed of the bubble in the pipeline is approximately equal to the flow speed, we took the flow injection speed *v* as the bubble speed. If the bubble in the micropipeline is in the form of an air embolism, we can take the microtube diameter *D* as the bubble diameter *d*. The bubble volume *V* can be estimated simply as:

(3) $$V=\frac{\pi }{4}d^{2}v\Delta t$$

If the bubble is a sphere (vΔt<d), the volume *V* can be calculated with the parameter of the high-accuracy calibration.

### 2.2. The Ultrasonic Probe Structure

**Figure 1:**
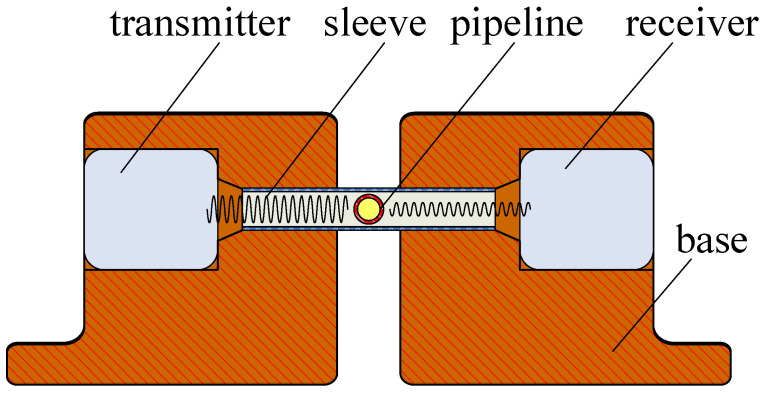
The structure of probe (section plane).

**Figure 2:**
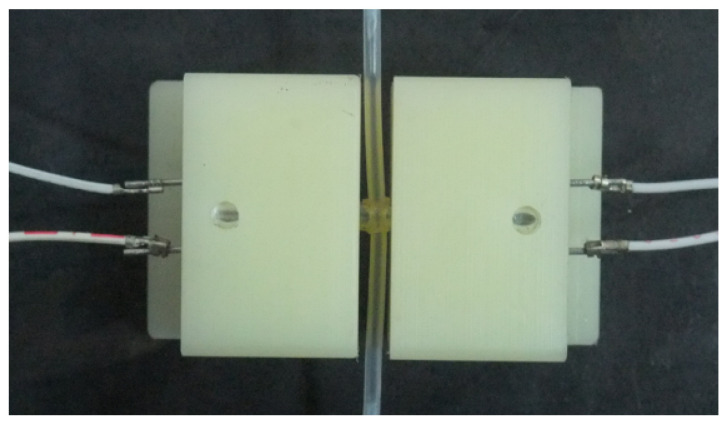
The prototype of the ultrasonic probe (top view).

The ultrasonic probe is a device that can transmit and receive ultrasound. Therefore, it is the key part of the microbubble-detection system. We selected the T40-160 and R40-160 piezoelectric ultrasonic devices (Audio Well Electronics Ltd., Guangzhou, China) as the transmitter and receiver. A higher ultrasonic frequency will detect smaller bubbles because of its smaller diffusion angle and better sonic wave directional characteristic, but the higher frequency signal has a lower anti-interference ability. In this paper, the ultrasonic frequency was 40 kHz, and the distance between the receiver and pipeline was no more than 10 mm. The structure of the probe is shown in Figure 1. We designed a sleeve joint between the transmitter and receiver. The micropipeline went through the side of the sleeve perpendicularly. The sleeve’s inner diameter was fit to the outer circumference of the micropipeline. This sleeve structure can lessen the sonic energy dissipation during the transmitting process. The prototype of the probe is shown in Figure 2, which is a top view.

In order to avoid contamination and facilitate the fit to the pipeline, we selected Delrin as the probe mounting material. Delrin possesses many excellent characteristics, such as acid and alkali resistance, wear resistance, good electrical properties, easy fabrication, low sensitivity to variations of the environmental temperature, etc. It is unfavorable if air exists in the installation gap of the parts, which would generate reflected wave clutter. We used PDMS to encapsulate the joints of the parts and utilized its flow-curing characteristic to eliminate air.

### 2.3. Design of the Circuit of the Microbubble Detection System

**Figure 3:**
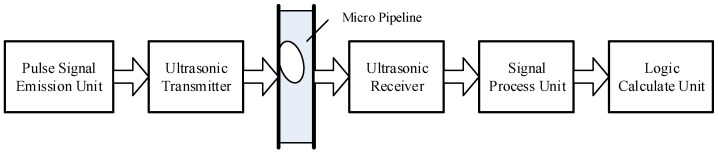
Flowchart of the bubble-detection system.

The circuit of the microbubble-detection system was composed of a pulsed-ultrasonic-signal-transmitting unit, an ultrasonic-signal-receiving unit, and a signal-processing unit. Its information flowchart is shown in Figure 3 In the pulsed-signal-emission unit, we adopted an SCM crystal system to launch square waves at 40 kHz, which had good integration and reliability, while we took advantage of the 74LS04 reverser to improve the driving capability. The AD620 (Analog Devices, Inc., Wilmington, MA, USA) operational amplifier, which can achieve an enlarged effect of 1000-times, was used as the signal-processing device of the ultrasonic receiver. When the signal was amplified, the noise component in the communication process was removed by the second-order filter circuit.

The key devices of the signal-processing unit were two AT89S51 SCMs (Atmel Corporation). The ADC chip was the AD7810 (Analog Devices, Inc.), whose upper and lower biases of the amplitude of the voltage were −0.4 V and +0.4 V. The storage unit was the CY7C132 (Cypress Semiconductor Corporation, San Jose, CA, USA). SCM 1 was responsible for the AD conversion and deposited the conversion results into the storage unit. SCM 2 was used to read the data of the storage and obtain the amplitude changes of the ultrasonic signal. Then, it could calculate the time that the bubbles took to go through the pipeline and the volume of the bubbles by comparing the voltage peak size. It could send messages to the PC, such as the magnitude of the voltage, the interrupt time, and the bubble volume. In the detection process, the energy attenuation of the ultrasonic signal within the bubbles was influenced by the temperature. In the experiment, we mounted a DS18B20 (Dallas Semiconductor, San Jose, CA, USA) intelligent temperature sensor to measure the temperature and fulfill the compensation calculation.

## 3. Experiments

**Figure 4:**
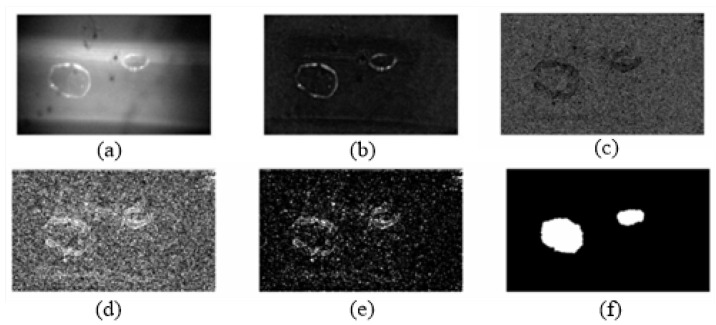
The processing of the bubble image. (**a**) Original image. (**b**) Background removed and brightness enhanced. (**c**) Edge detection. (**d**) Mathematical morphology expansion. (**e**) Mathematical morphology corrosion. (**f**) Bubble-edge-detection results.

**Figure 5:**
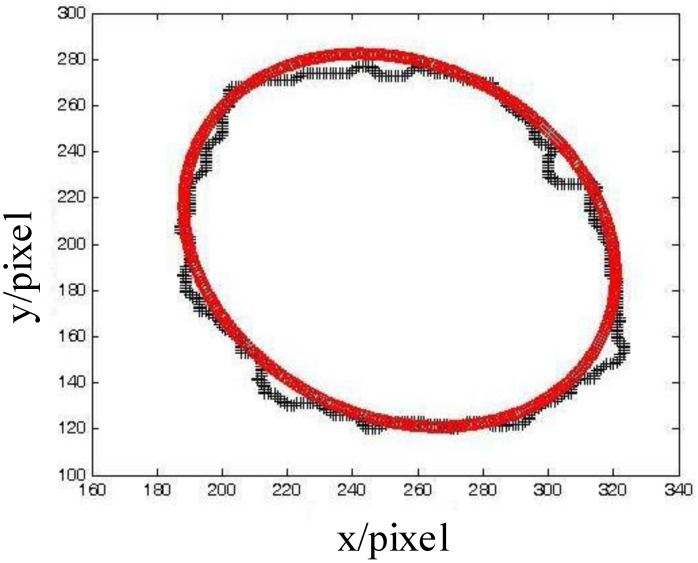
Ellipse fitting of the bubble.

In our developed microbubble-detection system, the signal processing was so complex that many factors could affect the measurement precision, such as the accuracy of the A/D conversion, the modulation enlargement, the shaping filter circuit, etc. In the experiment, we took advantage of the digital-image-processing technology to calibrate this system accurately. First, distilled water was injected and aspirated into a 1.5 mm-diameter transparent pipeline repeatedly via a syringe pump in order to produce bubbles randomly. The syringe pump was the CARVO XL-3000 (Tecan Group Ltd., Mannedorf, Switzerland). Then, we allowed the bubble–fluid to flow through the pipeline smoothly. We used a high-speed camera to acquire a bubble image before the bubbles went through the ultrasonic probe. The ultrasonic signal was calibrated using the data of the bubble characteristics from the images. Two formulas were used to calculate the volume of the bubbles. When vΔt > 1.5 mm (diameter of the pipeline), the bubble volume was calculated with a cylinder; when vΔt < 1.5 mm (diameter of the pipeline), the bubble volume was calculated with a sphere volume. Figure 4 shows one of the serial images and its calculating process. There are two bubbles in the image. Combining the mathematical morphology algorithm and edge-extraction algorithm, the image was enhanced by removing the background, and the bubble edges were extracted. An ellipses was drawn to fit the bubble outline in order to estimate the bubble volume. Figure 5 shows the ellipse-fitting result of the larger bubble projection in Figure 4.

Then, the image pixels were converted to the real size according to the camera parameters. The bubble projection area was equal to the ellipse area. The bubble volume was calculated with a sphere volume, the projection area of which was equal to the ellipse area. The bubble projection outline in Figure 4 was converted as follows: The long axis of the fitting ellipse was 0.649 mm, and the short axis was 0.527 mm. The bubble volume was calculated as 1.158 mm${}^{3}$. When the bubble was captured by the camera, we recorded the interrupt time of the ultrasonic detection system and the voltage signal from the ultrasonic probe. According to the bubble volume, which was calculated by the imaging system, we could calibrate the ultrasonic detection system. In the experiment, many bubbles of different sizes were detected and measured. Five bubbles of similar sizes were chosen to form a combination of sizes, and finally, ten sets with different sizes of bubbles were used for the calculation and analysis. The bubble size from the image was compared to the ultrasonic voltage signal. The time for bubbles to pass through the probe (Δt) was recorded.

## 4. Results and Discussions

Table 1 shows one bubble for each size. The experimental results of the image-processing method and the ultrasonic method are included. The serial numbers represent 10 different sizes of bubbles. When the volumes of the bubbles were 1.158 μL and 2.202 μL, the interrupt time of the ultrasonic detection system was zero. When the volumes of the bubbles were 2.832 μL, the voltage that came from the receiver of the ultrasonic detection system changed. However, the interrupt time of the system was still zero due to the error of the AD conversion and timing control of the AT89S51.

**Figure 6:**
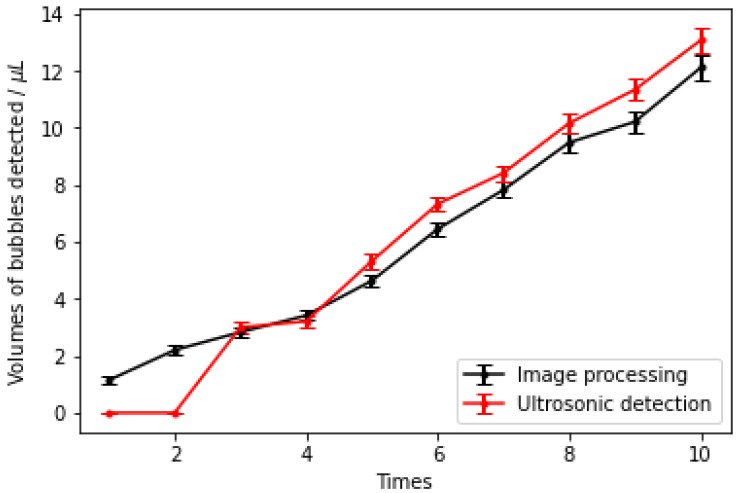
Comparative experiments of the image-processing and ultrasonic-bubble-detection system.

To avoid these errors, we determined the threshold of the ultrasonic detection system to be 3 μL. When the volumes of the bubbles were less than 3 μL, the voltage that came from the receiver of the ultrasonic detection system changed, and the interrupt time was zero. According to the experimental data, as long as the bubble volumes exceeded 2.832 μL, there was a significant change in the signal of the ultrasonic detection system. Therefore, the detection threshold of the bubbles was 3 μL. The results shown in Figure 6 also showed that the detection results had a small deviation from the image-processing results. The maximum deviation was 14.88%, and the minimum deviation was 5.94%.

In order to verify the effectiveness of the method under different parameter conditions, a sample-adding experiment was designed. That is, a given volume of liquid sample was added to a target vessel using a sample-adding system, which is usually a syringe pump. The volume of the liquid sample was compensated by measuring the volume of bubbles during the whole process using the proposed bubble-detection method. In the experiment, the bubbles were generated in the tube using the same method as above. Then, a 4 mL volume of the liquid was added using the sample-adding system via the tube. According to the volume of bubbles detected by the bubble-detection system, an additional corresponding volume was added to compensate the sample-adding system. Two sample-adding rates were used, i.e., different speeds of the liquid and bubbles when performing the bubble-detection method. After adding the sample, the liquid was weighed before and after the bubble detection using an electronic balance. Ten experiments were performed for each speed. The results are shown in Table 2 and Table 3.

From the results, it can be seen that the average volume of detected bubbles was 84.53 µL at a flow rate of 0.667 mL/s. The accuracy of the sample-adding system was improved by 1.876% after compensation by the bubble detection method. At flow rate of 0.067 mL/s, the average volume of detected bubbles was 73.48 µL, and the accuracy of spiking was improved by 1.363% after compensation. When the flow speed became slower, the bubble generation decreased. Regardless of the amount of bubbles generated, the proposed method can effectively detect the volume of bubbles and compensate the sample-adding system.

## 5. Conclusions

In this paper, we applied the ultrasonic-detection technique to the detection of bubbles in biological microsamples and developed an ultrasonic-bubble-detection sensor with the features of good applicability, easy manufacturing and installation, and high precision. We calibrated the bubble-detection system by the machine-microvision-measurement technology and determined the detection threshold of the bubble volume. The D/A conversion precision of the microcontroller and the interrupt timing error were the key parameters that affected the detection limit of the system.

## Figures and Tables

**Table 1:** The detected bubble volume of the bubble-detection system.

No.	Bubble Volume from Image Processing (μL)	Probe Amplitude (V)	Bubble through Time (s)	Bubble Volume from Ultrasonic Detector (μL)
1	1.158	0.412	0	0
2	2.202	0.392	0	0
3	2.832	0.386	0	3.000
4	3.415	0.354	1.56	3.212
5	4.636	0.345	2.49	5.326
6	6.449	0.322	2.48	7.328
7	7.821	0.287	4.07	8.414
8	9.483	0.245	6.11	10.162
9	10.212	0.234	6.39	11.340
10	12.11	0.212	7.20	13.071

Note: temperature: 20 °C; flow rate: 0.667 mL/s; the sample liquid is distilled water with a density of 0.9982 mg/mL.

**Table 2:** Results of the sample-adding experiments using the bubble-detection method for compensation (flow rate: 0.667 mL/s).

No.	Liquid Mass without Compensation (g)	Bubble Volume (μL)	Liquid Mass with Compensation (g)
1	3.8946	105.2	3.9857
2	3.8953	109.6	3.9921
3	3.8813	84.2	3.9611
4	3.8960	98.1	3.9810
5	3.8903	101.2	3.9803
6	3.8928	92.3	3.9728
7	3.8873	63.2	3.9413
8	3.8876	63.1	3.9425
9	3.8829	68.2	3.9429
10	3.9012	60.2	3.9536

Note: temperature: 20 °C; the sample liquid is distilled water with a density of 0.9982 mg/mL.

**Table 3:** Results of the sample-adding experiments using the bubble-detection method for compensation (flow rate: 0.067 mL/s).

No.	Liquid Mass without Compensation (g)	Bubble Volume (μL)	Liquid Mass with Compensation (g)
1	3.9627	87.2	4.0416
2	3.9521	54.2	3.9914
3	3.9462	94.5	4.0293
4	3.9755	65.3	4.0127
5	3.9205	108.3	4.0165
6	3.9737	58.6	4.0104
7	3.9436	58.9	3.9842
8	3.9236	99.6	4.0065
9	3.9421	56.3	3.9649
10	3.9436	51.9	3.9711

Note: temperature: 20 °C; the sample liquid is distilled water with a density of 0.9982 mg/mL.

## Data Availability

The data presented in this study are available upon request from the corresponding author.
